# Why do we Need to go Digital? Process of Developing an Online Facilitator Training Platform for a Global Family Skills Programme for Drug Use Prevention

**DOI:** 10.1007/s10935-023-00754-y

**Published:** 2023-12-21

**Authors:** Karin Haar, Mara Urlicic, Aala El-Khani, Giulia Martinelli, Wadih Maalouf

**Affiliations:** 1 United Nations Office On Drugs and Crime (UNODC), Wagramer Strasse 5, 1400 Vienna, Austria; 2 United Nations Office On Drugs and Crime (UNODC), Wagramer Strasse 5, 1400 Vienna, Austria

**Keywords:** Strong families, Family skills programme, Online facilitator training platform, Online course, Drug prevention

## Abstract

Strong Families is a programme developed for families living in challenged or stressful settings to prevent poor mental health and developmental outcomes, violence, and substance use. Facilitators are conventionally trained in person over two full days, by experienced international trainers. During the COVID-19 pandemic and due to travel restrictions, we developed an online course to deliver the content of the training manual electronically, with videos explaining the most difficult exercises, note taking functions and click and reveal activities to check their understanding. We further blended synchronous and asynchronous course formats to accommodate facilitators’ different time zones and work schedules. We tied two educational theories (Malcom Knowles theory of andragogy and Blooms taxonomy) into the Strong Families online course, to ensure learners are easily able to understand content, remember it and implement the gained skills within their communities. The aim of this paper is to discuss the process of the development of the Learning Management System and the Strong Families online course, as well as its benefits, key tools and essential considerations for replication through the UNODC multi-country and inter-disciplinary experience in digitalizing the Strong Family skills prevention tool to support other institutions interested in such a process, including in anticipation of future similar circumstances. To date, our online course has been made available in 10 languages, benefitting facilitators from 11 countries and the respective beneficiary families. Further impact evaluation, fidelity of implementation during national scale up and return on investment of integration of blended-learning concepts still need to be assessed.

## Introduction

Primary caregivers play a key role in preventing psychological morbidity in children living through prolonged periods of stress, hence preventing poor mental health and developmental outcomes, violence, lower educational attainment and substance use (Tol et al., 2013). Interventions that encourage trusting, sustainable and enrichening relationships between caregivers and children in their early years have shown to reduce drug use, child maltreatment and childhood aggression (WHO, 2009). Such family skills programmes combine knowledge transfer on parenting, in addition to skill building and further enhancing competencies, as well as providing reciprocal support (Barry, 2001). These primary prevention programmes have been recognised and recommended as evidence-based multi-outcome programmes through a number of guidance documents and inter-institutional collaborations such as the UNODC WHO International Standards on Drug Use Prevention (UNODC & WHO., 2018), the INSPIRE initiative to end violence against children (WHO & Cdc, 2016), the Violence Prevention Alliance (Wessels et al., 2013; WHO, 2009) and the Helping Adolescents Thrive initiative (WHO & Unicef., 2021).

The United Nations Office on Drugs and Crime (UNODC) has been implementing evidence-based family skills prevention programmes in low- and middle-income countries globally since 2010 (Campello et al., 2016). In 2017, the Strong Families programme was developed by UNODC, and first piloted in Afghanistan (Haar et al., 2020). Strong Families is designed to improve parenting skills, child well-being and family mental health in families living in stressful settings (Sandler, 2001). It is a selective, evidence-informed prevention intervention designed for families with children aged between eight and fifteen years living in stressful settings. Families in such context are generally invited using an opportunistic ‘universal’ approach, in which research assistants or facilitators recruit families without targeting any particular risk group. The programme was designed to be brief and is delivered to groups of families over 3 weeks. In week one, a group of around 12 caregivers meet for the one-hour (1-h) caregiver pre-session to explore their challenges and to develop ways to better deal with stress. In week two, the same caregivers discuss the means of showing love while enforcing limits and listening to children. In parallel, the children learn in another room how to deal with stress. After these 1-h parallel sessions, all caregivers and children immediately meet in one room for another 1-h family session where they practice positive communication and are encouraged to practice stress relief techniques together. In week three, parents learn to encourage good behaviour and discourage misbehaviour, while children explore rules and responsibilities and think about future goals in addition to the important roles their caregivers play in their lives. In the final family session, caregivers and children learn about family values and practice sharing appreciation to each other. More information on the content of the sessions of the Strong Families program can be found on the UNODC website (UNODC, 2019).

**Fig. 1:**
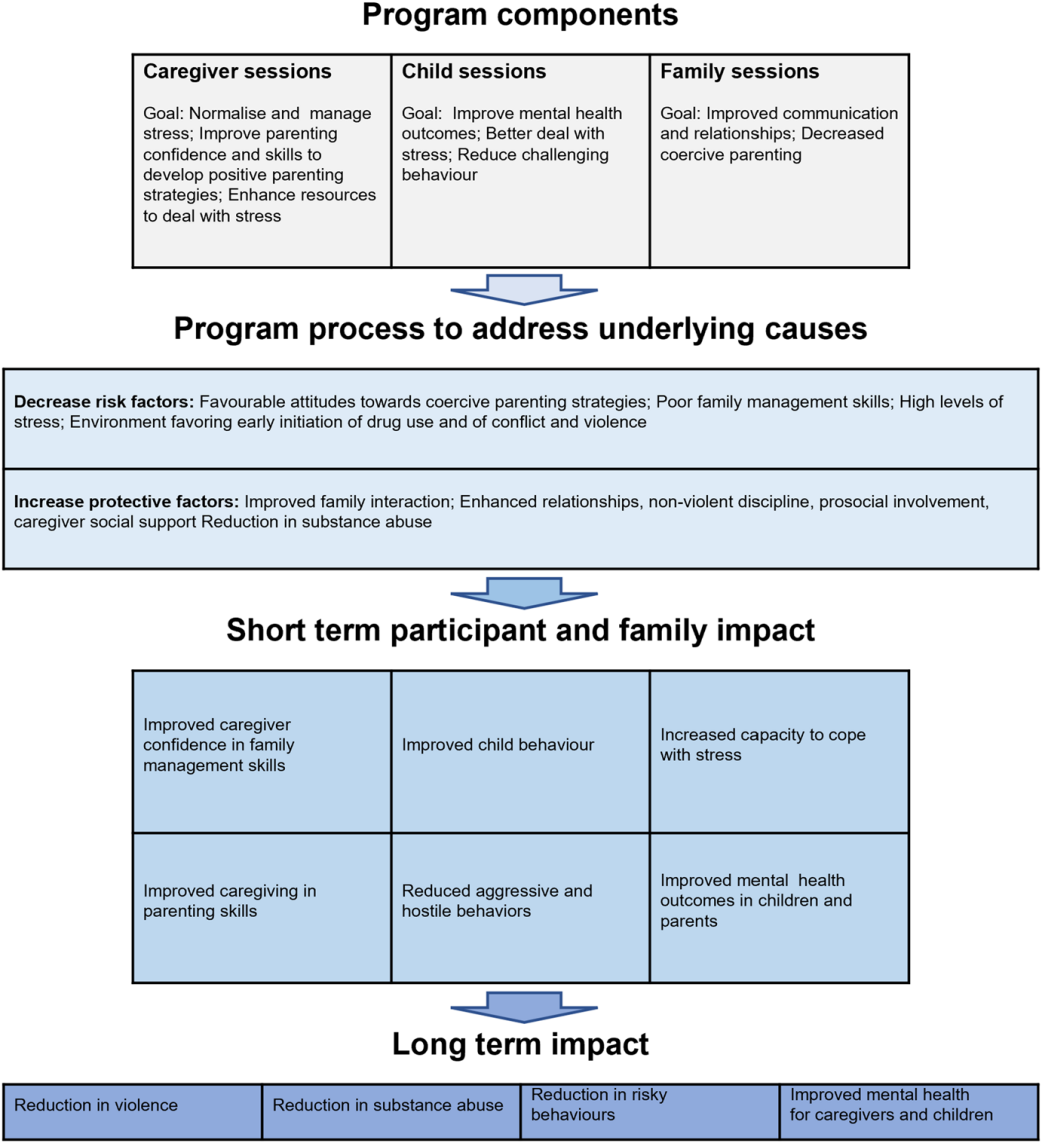
Logic model of strong families (UNODC, 2019)

The goal of the Strong Families programme is to support families in both recognising their strengths and skills, and further building on their strengths by sharing their challenges, as well as the things that work for them. Three overarching theories shaped the programme component and logic model (Fig. 1) of Strong Families, the biopsychosocial vulnerability model (Kumpfer et al., 1990), the resiliency model (Richardson et al., 1990) and the social learning theory (Bandura & Walters, 1977), which have been described in detail as well as their relevance in challenged settings previously (El-Khani et al., 2022; Haar et al., 2021).

Since its piloting, Strong Families has been implemented with families in a number of different countries and settings. It has been shown feasible to be implemented in low-resource and challenged setting and successfully delivered by trained lay facilitators, as shown in single-arm implementation studies in Afghanistan (Haar et al., 2020) and Serbia (El-Khani et al., 2021). In addition, a comparative trial proved its impact in families living in Iran in 2019/2020 (Haar et al., 2021). Strong Families was developed as a programme ensuring easy implementation within limited resource settings. Hence, its requirement of delivery materials to families as well as formal educational background of facilitators is minimal (UNODC, 2019). The only requirement is that facilitators have access to families and that they show an interest in delivering them the programme. Facilitators are usually trained by one or two international master-trainers through a two-day (16-h) interactive in person workshop where they also practice its delivery with their peers role-playing real families. Facilitators receive a training manual, which they are supposed to use when working with families. As soon as possible, following the in-person workshop, they are prepared to reach families and practice the implementation and revert back to trainers with feedback to complete their theoretical training through a practicum. Strong Families was designed to be resource “light”, requiring minimal resources for implementation. Facilitators will need to organise two rooms for families to hold the sessions (in the past, classrooms or community centres have shown to be successful (El-Khani et al., 2021; Haar et al., 2020, 2021)), and some minimal additional materials, such as paper and pens, a chalkboard or flipchart and some ropes and balloons. All training sessions can be conducted without electricity, internet, or any other tools, and can also be held outdoors, as this was the case during COVID-19 physical distancing measures.

## Benefits of Online Training, Especially During COVID19, and The UNODC Experience

One of the greatest advantages of online learning is flexibility in terms of time and place. Learning content is usually made available in short self-paced modules and can be paused at any time.

Another advantage of online learning is reduced financial costs. Online education is far more affordable as compared to face-to-face trainings. Furthermore, all the learning materials are available online, thus creating a paperless learning environment which is more affordable, while also being beneficial to the environment.

The COVID-19 pandemic has caused an unprecedented crisis in all areas. In the field of education, this emergency has led to the massive closure of face-to-face trainings of educational sphere in order to prevent the spread of the virus and mitigate its impact. In the case of global training, travel restrictions posed additional major restrictions.

Suspension of face-to-face trainings in many countries during the COVID-19 crisis has given rise to three main areas of action: the deployment of distance learning modalities through a variety of formats and platforms; the support and mobilization of education personnel and communities; and concern for the health and overall well-being of trainees and students (UNESCO, 2020).

One UNODC example of a global response to address a unique challenge, is that of police officers facing enforcing law and order during the COVID-19 pandemic, the first ever online training course for Kenya’s National Police Service (NPS) was launched on 29 July 2020. The training is the result of collaboration between the NPS, European Union and UNODC, and comprises seven e-learning modules that police officers can complete at their own pace on a computer, tablet or smart phone. Among the topics covered are the use of force, human rights approaches to crowd control, handling of sexual and gender-based violence cases, bail and bond, and how to deal with special interest groups such as persons with disabilities and children in conflict with the law.

Another UNODC example of implementing online training in response to the pandemic is offered by the Countering Terrorist (CT) Travel Programme, that delivered its first online training course on the detection of terrorists and their travel movements using travel information in October 2020. This was the first blended learning training course delivered by the United Nations Office of Counter-Terrorism (UNOCT). The course was hosted by the UNODC e-learning platform, which gave course participants the opportunity to watch pre-recorded lectures at their own pace and even come back to the information once the course was finished.

In these examples, online learning proved to be a helpful tool in reaching communities during the pandemic. Naturally, the application of online learning must also be considered within the context of potential barriers to access, such as low streaming bandwidth, intermittent or no internet access, government blocking of certain websites, lack of suitable electronic devices, limited understanding of learners on how to use technology.

The Need for Strong Families to go Digital During COVID19.

To tackle the COVID-19 pandemic, countries have implemented various public health measures, from lockdowns to social distance maintenance, temporary school closures, home-offices, and more. Apart from the health-related impacts of the pandemic, and its economic sequelae, the measures carried a heavy burden on families, leaving caregivers overwhelmed and unsure how to best respond to their children’s anxieties, worries, behaviours, and needs. Social isolation has been found to increase stress and may have harmful effects on both mental and physical health (Cacioppo et al., 2010). Stress and compromised parenting often place children at risk of violence, abuse, or neglect (Beckerman et al., 2017).

Recognising that existing family skills interventions that called for grouping of families during participation were not always possible, UNODC, joined by many agencies and international organizations, began rapidly developing a number of light-touch self-read parenting resources to enhance parenting skills and family harmony. These were translated into more than 50 languages (UNODC, 2020) and distributed through national partners and stakeholders. Implementation of Strong Families was feasible with COVID-19 restrictions (such as physical distancing, disinfection and mask wearing and more) in countries where implementation was initiated pre-pandemic, but pandemic measures (especially travel restrictions of international trainers) made it difficult to train new facilitators in some countries for scale up or to engage with new countries.

Despite all efforts, a need for an online digitalised learning platform was identified as essential to continue the global implementation of Strong Families. Beyond the self-reading parenting tips availed, this would allow for more facilitators to be able to engage with families in more interactive ways to develop skills needed within families.

The implementation of the Strong Families online course, capitalizing on UNODC existing experience, carried many potentials. It allowed for reaching a wider target audience of facilitators, which are characteristic of the benefits of online learning. Particularly the online course enabled inclusivity of the learner groups such as those geographically dispersed, limited time and/or resources to travel; those with other commitments hampering attendance of a course on a particular day on a fixed timetable; temporary workers such as consultants, part-time workers and self-employed workers. It also allowed access to areas in conflict and post-conflict zones, where there was restricted movement for security reasons as well as resolving problems with real-time communication (e.g. not native-English speakers) (FAO, 2021). Further, the format of the online course has the benefit of being available to learners at all times. Learners have the opportunity to post questions on the course message board or review any module of the course during or after the running of the synchronous online course.

An additional advantage of the Learning Management System (LMS) (an online platform that hosts the course and allows for learner administration and course evaluation) was supporting the development of a pool of master trainers. These were national trainers, trained by the global master trainers using the LMS and then on completion, would implement three cycles of the programme with families in their communities, then utilize the platform themselves to train other facilitators. Such a platform is as such key for strengthening the national training infrastructure in a country through echo trainings as well as to provide a backup booster training infrastructure for facilitators during implementation, supporting as such scale up and fidelity potential (Beckerman et al., 2017; Cacioppo et al., 2010; UNODC, 2020).

This paper describes the process of the Learning Management System (LMS) and the Strong Families online course, as well as its benefits, key tools and essential considerations for replication.

The Concepts Behind the Development of the Strong Families Online Platform.

The Strong Families online course aims to impact learners’ knowledge, skills and attitudes. In order to achieve an effective learning experience, several learning theories tied-in into the format of the Strong Family course. Such learning theories are crucial to achieve an effective learning experience (Teaching Excellence and in Adult Literacy (TEAL) Center. Adult Learning Theories. 2011) and to ensure learners are easily able to understand content, remember it and implement the gained skills within their communities. Two well-established educational theories form the basis of this course: Malcom Knowles theory of andragogy and Blooms taxonomy.

Malcom Knowles theory of andragogy is based on five principles (Cochran et al., 2016). First is the adults’ self-concept, whereby adults seek to be independent and self-directed in acquiring knowledge. Secondly, their motivation stems from internal interests rather than external rewards. Thirdly, given their age, adults have accumulated an array of experiences and strive to incorporate their existing knowledge into new learning experiences. Furthermore, adults present a readiness to learn, which is solution oriented. Finally, the fifth principle is that they are motivated by seeing an immediate and practical impact on their daily tasks. There are several ways in which the theory of andragogy was applied in the Strong Families online course, such that all learners were granted access to entire modules at a time, to allow learners to independently visit each section and complete multiple-choice questions (MCQs) and assignments when they felt confident that they had fully understood the module. Moreover, learners were asked to share their personal experiences and expectations as they progress through the activities in the webinars (e.g., “what reactions have you seen in children that could be from added stress?”). In addition, the structure of the online course reflects the structure of the face-to-face trainings the learners will facilitate in their communities. Thereby, they have a direct link to how the online course translates into their daily practice. Also, the online course is scheduled to take place imminently before the learners go into their communities to facilitate the face-to-face training for caregivers and children. Finally, the learners are given a platform to gather knowledge and share with others, rather than being enticed with external rewards for completing the training.

**Fig. 2:**
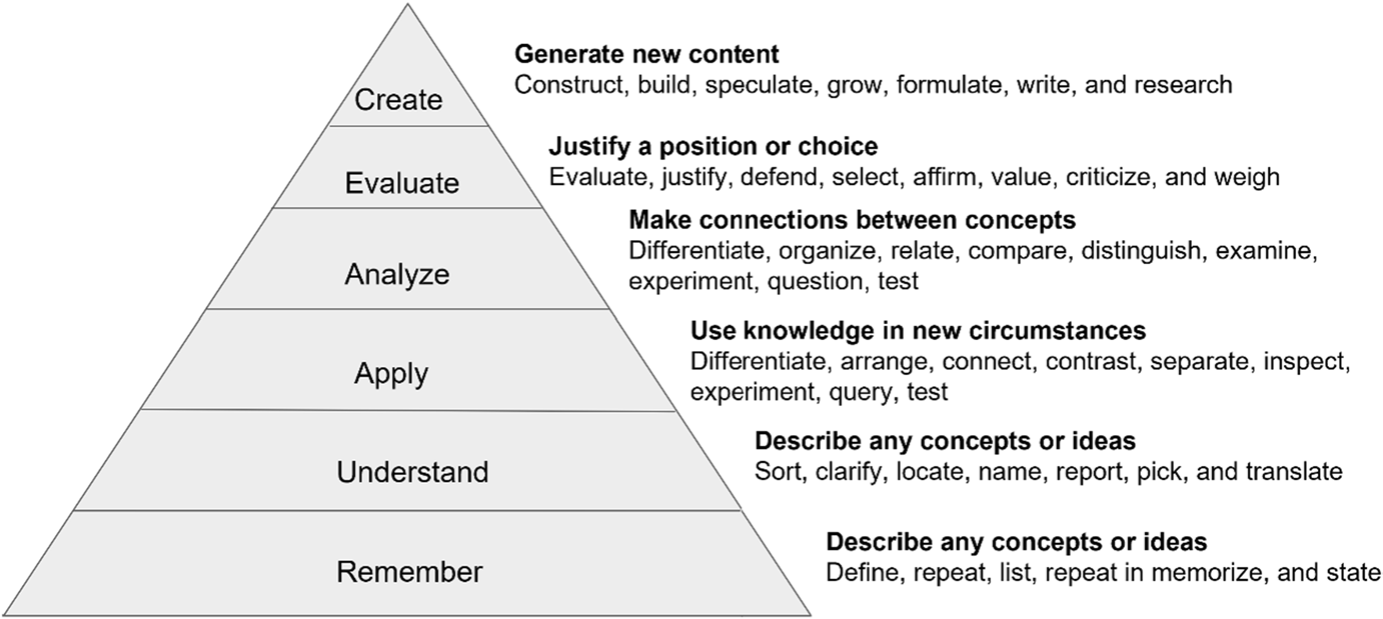
Bloom’s taxonomy (Armstrong, 2010)

As for the Blooms taxonomy theory (Fig. 2), this is a cumulative hierarchy of six cognitive domains, which reflect the level of complexity required to gain a certain competency. At the bottom of the hierarchy, learners can simply recall information, while at the highest level of complexity, learners are able to create independent work based on their thorough understanding (Krathwohl, 2002):

The Strong Families online course aims to mainly support learners in remembering, understanding and applying the activities of the programme when working in their communities. Given that the programme is also heavily based on transmitting a set of values, the webinars, flip chart activities and assignments are essential in ensuring the learners utilize the higher cognitive domains and internalize the principles of the Strong Families programme.

The certification the learners obtain from participating in this online training is then built upon by obtaining a further certification upon implementing the learnt programme in their communities. This ensures learners gain valuable knowledge, skills and attitudes during the online training, which they are immediately able to apply to their work and thereby strengthening comprehension and practice.

## Key Tools in the Strong Families Online Course

### Simple Learning Resources

Simple learning resources are non-interactive, such as documents, content presented in the LMS and animated videos. The Strong Families online course adapted the face-to-face training manual to incorporate simple interactivity, of playing videos and allowing text input fields. Simple learning resources were used in the creation of the Strong Families online course to ensure the core materials would be accessible to all learners participating in the course, even if they live in a region with limited or intermittent bandwidth. Thereby the risk of slowing application performance or reduced user productivity could be avoided. A further reason for relying on simple learning resources was to ensure learners of all computer skills and technical expertise were able to follow the course. To support learners further, at the start of the course a frequently asked questions (FAQ) section was provided to answer common questions.

Overall, learners are expected to spend approximately 15–20 h with the Strong Families online course, depending on their ability to read and practice for the assignments.

### Quizzes

Quizzes (also called tests, assessments, or knowledge checks) are an essential component of e-learning. They can be integrated into an online course or be provided as stand-alone learning components. Tests help to assess learners’ progress, as well as the effectiveness of learning. They also have the potential to increase learners’ engagement and to support the learning process through the provision of personalized feedback. Each module of the Strong Families online course included a quiz section of 5–10 MCQs. This provided a quick knowledge check of whether the learner had fully comprehended this segment. It also allowed course tutors to see whether an individual or perhaps several persons in the group were excelling or struggling with a particular aspect of the course.

### Live Webinars

During the webinars, two course master trainers instructed the learners remotely, and in real time (through interpretation when needed), using a combination of materials (e.g. PowerPoint slides, videos and digital white boards). This aspect of the online course was most similar to the face-to-face trainings that had taken place onsite. The content of the webinars was interactive and ensured key messages presented throughout the course were clearly understood by learners. Regardless of which professional background the individual learners came from, they all were given an opportunity to demonstrate their application of the obtained knowledge and skills in the subject.

### Synchronous and Asynchronous E-Learning

Synchronous learning is a form of education that occurs at the same time, but not in the same place whereas asynchronous learning occurs not only in different locations, but also at different times (Great Schools Partnership, 2014). Both synchronous and asynchronous settings bear their own set of advantages and disadvantages (Fabriz et al., 2021). The Strong Families online course webinars were available for live synchronous participation as well as to be reviewed on-demand as a recording of the webinar. This made the course available to learners who may have missed a webinar (e.g., due to illness) as well as supporting all learners in reviewing materials already covered in the course. It was ensured that for synchronous participation, all learners were in the same time zone and thereby able to join.

### Recording of Homework—Use of Video Tools

At the end of each module, learners were provided the opportunity to reflect on learnt content and demonstrate their understanding by submitting a relevant assignment. These assignments may include, completing a simple activity that they would be asking caregivers or children to complete when they facilitate the face-to-face trainings with them. Alternatively, many assignments required collaboration between learners by running through a complex activity from the face-to-face training and submitting a recording of this for review. All submitted assignment were reviewed by the online course facilitators and feedback was provided in order to pass the assignment.

## Essential Considerations

### Internet Access

LMS are key components of distance learning strategies. The services of LMS include supporting the delivery of lessons, hosting and managing learning materials, and supporting communication. The bandwidth of these platforms needs to be upgraded based on an estimate of the increased number of simultaneous visitors. Courses and content of the platforms should be intentionally designed to enable inclusive accessibility. The UNODC Global eLearning Programme encourages and supports community-based access initiatives, educational networks, and development initiatives that enable diverse models for access and use. Furthermore, it has increased its capacity and user-friendliness in response to COVID-19, making its course catalogue more accessible and inclusive.

### Security

As an internet-based learning method, online learning depends on the internet for its execution (Mohd Alwi & Fan, 2010). In this regard, it must be considered that security threats might occur on the internet. Consequently, the e-learning environment is inevitably exposed to constant security threats, risks, and attacks. Previous literature indicates that security has three basic requirements: confidentiality, integrity, and availability (Adams et al., 2003). To avoid attacks upon the eLearning environment, controlling access is paramount. The UNODC Global eLearning Programme addresses the security threats by focusing on the platform access management (identity of users). One of the ways to do this is via the authentication process. UNODC Global eLearning Programme recommends an authentication process to identify a legal user process. Furthermore, the platform provides users with single sign-on authentication to all authorized web applications and web resources.

Confidentiality refers to the protecting of sensitive information from being accessed by unauthorized persons and the absence of unauthorized disclosure of information (Serb et al., 2013). To protect personal information, security safeguards such as authentication and encryption were implemented. Integrity refers to “the protection of data from intentional or accidental unauthorized changes” (Serb et al., 2013), hence access control was implemented. Availability means the readiness for correct service (Weippl & Ebner, 2008) and our LMS was able to be accessed by authorized users whenever needed.

### Single or Group-Participation in Webinars

Based on the physical distancing measures in each respective country, some facilitators participated in the webinars in front of their computers at home, whereas others were able to group, and participate jointly in webinars. All of them were provided with the option to participate in the webinars, irrespective of their location and grouping. In addition, facilitators who grouped in one location, were able to practice the tasks discussed immediately, and to submit assignments soon after. In addition, they were given pre-prepared materials, such as flipcharts and printed materials by the organisers of the local webinars, and hence could practice immediately, while those participating individually from their homes were expected to prepare the materials themselves, according to the instructions in their manuals and supplementary materials.

Collaborative learning covers discussion forums, knowledge sharing in webinars as well as working together on completing assignments. This helped learners clarify and practice what they had learnt as well as empowered others in their abilities. Collaborative learning was a core aspect of the Strong Families online course as learners were often present in the same room when participating in the webinars as well as relying on each other’s assistance in completing homework assignments.

### Interpretation/ Translation of Tools and Software

To prepare the online course for various countries, the latest version of the training manual and supplementary materials needed to be translated, along with the text for the LMS, which included quizzes, assignments, evaluation- and data consent forms. The animated videos were then prepared accordingly and integrated into a pdf version of the manual that allowed integration of all sorts of digitally rich media into the document like visual weblinks, videos, audio, GIFs, files, and other documents (interactive pdf). Finally, all functionalities were tested in the respective country through our national/local counterparts. When translating content close attention was paid to creating streamlined documents which clearly indicate what text is to be translated, where to write the translations and finally, where these translations will appear in the online course. Keeping the effort of translations in mind, this online course relied heavily on the written word rather than audio sequences, which are harder to place accurately within videos. Given the scope of languages, it was further important to enquire the suggested font for each language and from there deduct which authoring tool would be able to provide this. For the webinars, simultaneous interpretation was organised to ensure smooth, fast and two-way communication.

### Process to Develop the Online Course

**Fig. 3:**
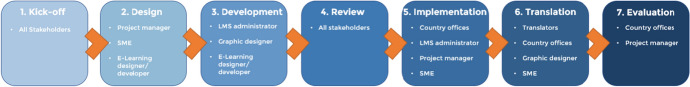
Process of developing the online course

The online course was set up within 4 months, from the initial kick-off meeting to implementation. The following stakeholders were involved in completing the course: project manager, subject-matter-expert (SME), online course developer, graphic designer, LMS administrator, translators, implementation partners in country offices and donors. The process of developing the course is detailed in Fig. 3.

The initial kick-off meeting was crucial to identifying a common vision and scope of the project. We defined the learner groups, need for the e-learning, content to be covered, roles of stakeholder’s timeline, budget and KPIS for evaluating the course. In the design phase the SME and project manager collated all materials to be used for the e-learning course. The online course developer created a storyboard based on the materials. This served as a blueprint of how the learners will engage with the content, how learners progress will be assessed and how to ensure all learners needs are met throughout. To ensure all stakeholders are content with moving forward, feedback was collected, and the storyboard updated accordingly.

In development phase the online course developer and graphic designer created the content, media and interactions. The LMS administrator supported the team in implementing the developed e-learning materials on the LMS. The alpha version of the online course was presented to stakeholders for feedback, resulting in the beta version, which was once more reviewed and updated. Ultimately, the online course was signed off by stakeholders as meeting the intent of the project.

During initial implementation the project manager liaised with country offices to coordinate training dates, participants and setting up their accounts on the LMS. The SMEs played a central role to delivering the trainings (particularly facilitating the webinars and marking assignments), while the LMS administrator supported learners with technical issues. Due to the promising feedback of the pilot implementation, the online course was subsequently translated into several languages through the respective country offices. The online course developer, graphic designer and LMS administrator implemented these translations into the various e-learning materials and LMS. Subsequently, the online course was reviewed by the country offices and feedback was incorporated, as needed. Finally, the translated online course was launched in collaboration with the project manager, SMEs and country offices as before.

This evaluation of the online course is planned during the coming months and will include quantitative and qualitative elements.

### Delivery of the Strong Families Programme Through Remotely Trained Facilitators

Up until July 2022, the online platform has so far been made available in 10 languages, benefitting 483 facilitators from 11 countries (Bolivia, Mexico, Bosnia & Herzegovina, Serbia, Cambodia, Thailand, Myanmar, Zambia, Turkmenistan, Iran and Afghanistan). Feedback from learners was received from five countries (Cambodia, Thailand, Turkmenistan, Zambia and Bosnia & Herzegovina) through self-completed questionnaires regarding materials & presentation, technical aspects of the platform, communication, organisation/structure of training, contents of the course, trainers and overall ratings. Families have already been reached through facilitators trained by the platform.

Data on child mental health, parenting practices, family adjustment skills and child resilience are currently being assessed as collected for further outcome evaluation as well as fidelity data from external and internal sources for additional process evaluation.

## Discussion

The Strong Families e-learning course has been well received by country offices and obtained positive feedback by learners. The course has already reached almost 500 facilitators, who will continue to reach many families globally. Nonetheless, there are a few elements that could be considered to further improve the course and its implementation.

Firstly, the Strong Families e-learning course is currently gaged towards desktop learning. While some elements (e.g., MCQs and animated videos) are also mobile friendly, other aspects of the course (e.g. webinars, interactive pdf, submitting of assignments) would need to be revised to be easily accessible through mobile phones (Burgstahler, 2021). For instance, the webinars could be broken into shorter more frequent sessions, pdf content could be posted directly within the LMS and assignments could be adapted to require less bandwidth when submitting.

Further, the webinars could be designed to allow for more interactions between learners to strengthen understanding and build the learners network in their communities. Some examples of making webinars more inclusive and interactive include, breakout groups, voting in a poll and shared virtual whiteboards (United Nations Institute for Training and Research (UNITAR) xxxx). Given that the initially developed Strong Families face-to-face trainings can resume following the COVID-19 pandemic, the Strong Families online course could be revised to support the onsite trainings. Examples of this blended-learning concept may include: (a) preparing learners ahead of the face-to-face trainings through the e-learning materials and activities, (b) supporting learners during the face-to-face trainings with materials to be used in the session or between training days, (c) following the face-to-face trainings with materials to review and build upon understanding gained during face-to-face training (FAO, 2021) or (d) scaling up the capacity nationally by training additional facilitators through national Master-trainers succeeding their graduation and successful implementation with families.

For the future, the e-learning content could become more accessible for populations with lower literacy capacities (or to generally reduce the volume of reading) by offering text-to-speech reading in the LMS, as well as voice-overs in the animated videos or scripts for webinars. However, to date, we have yet to receive such requests. When implementing the Strong Families programme, facilitators write on flipcharts, however in the future, an entire “language-free” course could be considered, utilizing only symbols and drawings, to be also more inclusive to illiterate families.

Finally, the Strong Families online course targets facilitators to teach them how to implement the programme in their communities. To provide direct support to the children and families in the communities, a mobile phone application is currently being developed to further extend the impact of the online course through targeted exercises for families to be repeated also after the engagement with the facilitators. Piloting of such a mobile phone application will provide feedback on the usefulness and feasibility for families globally.

Fidelity for those receiving Strong Families through the eLearning platform will need to be ensured, the research regarding such is currently ongoing. The main outcome will be to explore if facilitators trained through the online platform are able to deliver the programme as those who were trained through face-to-face training. Self-assessments, as well as reports from independent observers will guide us in this evaluation. Qualitative research with facilitators, as well as final beneficiaries (children and caregivers) will support us in improving the online platform and guide towards remote or blended learning options. In addition, we aim to compare the impact on families through conventionally and remotely trained facilitators.

It is also worth mentioning that while the eLearning platform was originally conceived to overcome the impediment of reaching facilitators during COVID19 travel restrictions, this platform can also be used as resource support for trained facilitators to refer to as a booster to their training to further ensure fidelity of implementation during implementation scale up.

Despite the negative impacts of the COVID-19 pandemic, we believe that it has accelerated digital developments, and changed people’s attitude towards online training. This has led to several positive effects and benefits. Relying on UNODC’s experience with eLearning module development on one end, and on UNODC’s experience in the development and implementation of Strong Families, we can now reach greater numbers of facilitators globally; we can reach them in remote or unsafe areas and provide them with fast and updated information; and more valuably we can have a better scale up potential of the programmes at national level while ensuring fidelity through consistent teaching, recording and repetition.

## Conclusions

While the online platform development of Strong Families was prioritized and accelerated due to the COVID19 pandemic travel restrictions, lessons learned during this online implementation reflected the feasibility, flexibility and value of such an exercise. The online modality of facilitators’ training allowed for strengthening engagements in countries where implementation was initiated prior to the pandemic and required strengthening during the pandemic when vulnerabilities were accentuating begging for such family skills packages. Moreover, it allowed for further countries to benefit from Strong Families at a time when meeting their demands was difficult. Despite the existing qualitative data received by facilitators of Strong Families trained through the platform, UNODC is currently in the process of further analysing the effectiveness of this platform through diverse blended-training modality to optimize the training experience of facilitators, while amplifying the chances of scaling up the application of Strong Families with fidelity on a national basis. In addition, the costs and cost-effectiveness of Strong Families implementation with families need to be assessed in the future.

Global interest for digital learning is on an all-time high, including in anticipation of any further epidemics. We definitely need to go digital, however such a process particularly as it concerns tools that were pre-designed to be implemented in person (as is the case with many other skill- and/or competency- development tools) is not as straightforward as it sounds. It needs to account for educational theories applied on learners, tools used, process of development, method of delivery as well as other considerations such as (internet access, security, mode of access of learners and more). The experience learned from this UNODC multi-country exercise, and the inter-disciplinary implication of different specialized professionals in the development of the content, coupled with UNODC existing experience, allowed for a lot of insight being generated during development and implementation. This carried a lot of lessons learned for many other institutions interested in such an exercise. Moreover, reflections on this experience allowed the generation of further thoughts on return on investment for such a digital modality particularly as it pertains to potentials for scale up of implementation of such packages while ensuring fidelity, cutting down on cost of international travel while ensuring national ownership of processes.

## References

[CR1] Adams A, Blanford A, Ghaoui C (2003). Security and online learning: to protect and prohibit. Usability evaluation of online learning programs.

[CR2] Armstrong P. (2010) Bloom’s taxonomy. Vanderbilt university center for teaching. https://cft.vanderbilt.edu/guides-sub-pages/blooms-taxonomy/. Accessed 25/05/2022.

[CR3] Bandura A, Walters RH (1977). Social learning theory.

[CR4] Barry M (2001). Promoting positive mental health: Theoretical frameworks for practice. International Journal of Mental Health Promotion.

[CR5] Beckerman M, van Berkel SR, Mesman J, Alink LR (2017). The role of negative parental attributions in the associations between daily stressors, maltreatment history, and harsh and abusive discipline. Child Abuse and Neglect.

[CR6] Burgstahler S. (2021). 20 Tips for teaching an accessible online course. University of Washington (UW). https://www.washington.edu/doit/20-tips-teaching-accessible-online-course. Accessed 25/05/2022.

[CR7] Cacioppo JT, Hawkley LC, Thisted RA (2010). Perceived social isolation makes me sad: 5-year cross-lagged analyses of loneliness and depressive symptomatology in the Chicago health, aging, and social relations study. Psychology and Aging.

[CR8] Campello G, Heikkila H, Maalouf W (2016). International standards on drug use prevention: tools to support policy makers globally to implement an evidence-based prevention response. Cambridge handbooks in psychology.

[CR9] Cochran C, Brown S (2016). Andragogy and the adult learner.

[CR10] El-Khani A, Haar K, Stojanovic M, Maalouf W (2021). Assessing the feasibility of providing a family skills intervention, “strong families”, for refugee families residing in reception centers in Serbia. International Journal of Environmental Research and Public Health.

[CR11] El-Khani A, Calam R, Haar K, Maalouf W (2022). Bridging the gap between the pressing need for family skills programmes in humanitarian settings and implementation. International Journal of Environmental Research and Public Health.

[CR12] Teaching excellence in adult literacy (TEAL) center. (2011). Adult learning theories. In: Teaching excellence in adult literacy (TEAL) center, editor. U.S. department of education, office of vocational and adult education (OVAE)

[CR13] Fabriz S, Mendzheritskaya J, Stehle S (2021). Impact of synchronous and asynchronous settings of online teaching and learning in higher education on students’ learning experience during COVID-19. Frontiers in Psychology.

[CR14] FAO. (2021).E-learning methodologies and good practices: a guide for designing and delivering e-learning solutions from the FAO elearning Academy. Rome: Food and agriculture organization of the United Nations

[CR15] Haar K, El-Khani A, Molgaard V, Maalouf W (2020). Strong families: A new family skills training programme for challenged and humanitarian settings: A single-arm intervention tested in Afghanistan. BMC Public Health.

[CR16] Haar K, El-Khani A, Mostashari G, Hafezi M, Malek A, Maalouf W (2021). Impact of a brief family skills training programme (“strong families”) on parenting skills, child psychosocial functioning, and resilience in Iran: a multisite controlled trial. International Journal of Environmental Research and Public Health.

[CR17] Krathwohl DR (2002). A revision of bloom’s taxonomy: An overview. Theory into Practice.

[CR18] Kumpfer KL, Trunnell EP, Whiteside H, Engs RC (1990). The biopsychosocial model: Application to the addictions field. Controversies in the addiction field.

[CR19] MohdAlwi N, Fan I-S (2010). E-learning and information security management. International Journal for Digital Society..

[CR20] UNESCO (2020). Office santiago and regional bureau for education in Latin America and the Caribbean, economic commission for Latin America and the Caribbean. Education in the time of COVID-19: OREALC/CEPAL2020

[CR21] Great schools partnership. (2014). Synchronous learning. In: The glossary of education reform, Portland, ME. https://www.edglossary.org/synchronous-learning/. Accessed 25/05/2022.

[CR22] Richardson GE, Neiger BL, Jensen S, Kumpfer KL (1990). The resiliency model. Health Education.

[CR23] Sandler I (2001). Quality and ecology of adversity as common mechanisms of risk and resilience. American Journal of Community Psychology.

[CR24] Serb A, Defta CL, Iacob NM, Apetrei MC (2013). Information security management in e-learning. Knowledge Horizons—Economics.

[CR25] Tol WA, Song S, Jordans MJ (2013). Annual research review: Resilience and mental health in children and adolescents living in areas of armed conflict–a systematic review of findings in low-and middle-income countries. Journal of Child Psychology and Psychiatry.

[CR26] United Nations Institute for Training and Research (UNITAR). (2020). Making online events more inclusive: UNITAR2020

[CR27] W UNODC. (2018). International standards on drug use prevention: second updated edition United Nations Office on Drugs and Crime, World Health Organisation Vienna

[CR28] UNODC. Strong families programme. 2019. https://www.unodc.org/unodc/en/prevention/strong-families.html. Accessed 04/01/2022

[CR29] UNODC (2020). Parenting under COVID-19. https://www.unodc.org/unodc/en/listen-first/parenting-under-covid-19.html. Accessed 03/05/2022

[CR30] Weippl ER, Ebner M. (2008). Security privacy challenges in e-learning 2.0. In: Bonk CJ, Lee MM, Reynolds T, editors. In *E-Learn: World conference on e-learning in corporate, government, healthcare, and higher education*; Las Vegas, Nevada, USA: Association for the advancement of computing in education (AACE); p. 4001–7

[CR31] Wessels I, Mikton C, Ward CL, Kilbane T, Alves R, Campello G (2013). Preventing violence: Evaluating outcomes of parenting programmes.

[CR32] WHO. (2009). Violence prevention: The evidence: Preventing violence through the development of safe, stable and nurturing relationships between children and their parents and caregivers. World Health Organization

[CR33] WHO, CDC, (2016). End violence against children, PAHO, PEPFAR, together for Girls et al. INSPIRE: Seven strategies for ending violence against children. Geneva: World Health Organization. Report No.: ISBN: 9789241565356

[CR34] WHO, UNICEF. (2021). Helping adolescents thrive toolkit: strategies to promote and protect adolescent mental health and reduce self-harm and other risk behaviours. Geneva: World Health Organization and the United Nations Children’s Fund (UNICEF)

